# Surgeon’s narcissism, hostility, stress, bullying, meaning in life and work environment: a two-centered analysis

**DOI:** 10.1007/s00423-023-03068-z

**Published:** 2023-09-04

**Authors:** Michael El Boghdady, Béatrice Marianne Ewalds-Kvist

**Affiliations:** 1 Guy’s and St Thomas’ NHS Foundation Trust, London, UK; 2 Equality and Diversity Officer, Association of Surgeons in Training, London, UK; 3 University of Edinburgh, Edinburgh, UK; 4 Stockholm University, Stockholm, Sweden; 5 University of Turku, Turku, Finland

**Keywords:** Surgeons, Hostility, Narcissism, Stress, Quality of work-life, Meaning of life, Bullying, Training

## Abstract

**Introduction:**

Disruptive physician behaviour can affect patients’ safety. If surgical trainees throughout higher education experience disruptive behaviour, impaired work-life may follow. Therefore, we aimed to study surgeons' level of narcissism (N), hostility, and stress in relation to their work environment and potential experience of bullying. We also scrutinized search for or presence of meaning in life.

**Methods:**

General surgeons in UK National Health Service from 2 hospitals participated with 3 levels of training: junior trainees (JT), senior trainees (ST), and consultants (CONS). Participants completed 52 VAS-formed questions plus demographics. Modified questionnaires were used for assessments of ‘hostility’, ‘narcissism’, meaning in life, quality of work-life, and bullying.

**Results:**

Altogether 33% of surgeons displayed narcissism and 22% could exhibit disruptive behaviour. By MANOVA significant differences between low, medium, and high narcissism groups were revealed in hostility (*p*<.01), perceived stress (*p*=.001), and presence of meaning in life (*p*<.05). Regression analyses explained hostility both by N-scale (p=.000) and ‘being bullied during training’(*p*=.009) but negatively by ‘presence of meaning in life’(p=.004). Surgeons’ perceived stress was explained both by N-scale (*p*=.000) followed by ‘seeing others bullied during training (*p*=.000) and negatively by ‘working extra days beyond schedule’ (*p*=.007). The presence of meaning in life was explained mostly by good beneficial stress (*p*= .000) but negatively both by ‘doing extra work beyond schedule’ (*p*= .016) and hostility (*p*= .003).

**Conclusion:**

Surgeons may exhibit disruptive behaviour in a challenging situation. The narcissim-scale was the best predictor of hostility and perceived stress. Being bullied during surgical training predicted hostility. Seeing others being bullied during surgical training predicted stress. Beneficial stress is explained best by surgeons’ experience of the presence of meaning in life.

## Background

Disruptive physician behaviour is known to put the patient’s safety at risk and to increase the danger of malpractice litigation. Hostile behaviour, usually called ‘disruptive behaviour’, has been witnessed in 77% of physicians in 102 hospitals in the USA [1]. This kind of troublesome behaviour in healthcare eventually affects staff turnover as well as patient care and could be associated with medical errors, leading to patient morbidity or mortality [1–4]. It has been found that certain surgeons ranked high on attributes preceding disruptive behaviour such as narcissism or malignant self-love, that is, displaying an underlying unwillingness to recognize or identify with the feelings and needs of others [5]. This unwillingness to use one’s affective capacity to respond with an appropriate emotion to another person’s mental state is distressing for the surrounding [6, 7]. It has been previously revealed that self-love can be frequent among surgeons [8].

If surgeons throughout their higher training are exposed to disruptive behaviour, the consequences for them are an impaired work-life and a modified experience of the presence of meaning in life. The Royal College of Surgeons of England has observed through its invited review service that inappropriate behaviours can have an impact on the standard of surgical care [9]. There is insufficient evidence to establish the extent to which patient harm is caused by disruptive behaviours, but it is accepted that there is a direct link between the two factors which may jeopardize patients’ safety and cause distress for health-care professionals [9–11].

Accordingly, we aimed to study surgeons' level of narcissism (N), hostility, and stress in relation to their work environment and potential experience of bullying. In addition, surgeon’s experience of searching for or presence of meaning in life was scrutinized as protective factors in order to explore their relation to disruptive behaviour, if present.

The following research questions were posed:

- Are surgeons inclined to display narcissism?
- If so, does surgeons’ narcissism reflect hostility and stress levels?
- Are surgeons exposed to bullying during their training?
- Which factors impact most surgeons’ search for or presence of meaning in life?
- Does surgeons’ level of training reflect in the experience of meaning in life?

## Methods

### Participants

Surgeons working in the National Health Service (NHS) in the UK from two London hospitals were included. Participants were general surgeons. Trainee surgeons comprising junior trainees (JT) also known as house officers or those who are in the core surgical training and senior trainees who are in the higher surgical training pathway (ST), as well as consultants (CONS) were included in this study. The data collection was performed from October 2021 to June 2022.

### Questionnaires

Participants were asked to complete 52 questions. Questions from 5 validated inventories were inserted in the questionnaire in the forms of the visual analogue scale (VAS) from 0 to 60 mm indicating Disagree to Agree. The questions were reliability tested with Cronbach’s alpha and items were deleted until a minimum alpha of .70 for each cluster of questions was received.

### Demography

Participants’ age and 9 categorical variables in the form of gender, if they have children yes/no, level of training, surgical specialty, supervision, relaxation, extra work yes/no, work type, and extra work besides the main job were studied.

### Hostility

We included 12 items from Buss and Perry (1992) Aggression Questionnaire (BPAQ) [12] to measure the factors, verbal anger and hostility. Out of 12 items, 10 contributed to a Cronbach’s alpha of .788

### Narcissism

A total of 16 questions were selected and reworded from Narcissistic Personality Disorder (Campbell, Baumeister 2006) (NCB) [13] merging narcissism (4 items), narcissistic shame (2 items), entitlement (2 items), and need for admiration (2 items). The scale with 10 items added up to Cronbach’s alpha .777

### Meaning in life

Meaning in life questionnaire—Short-form (MLQ-SF) by Steger et al. (2006)—is a self-rating measure with an internal consistency by Cronbach’s alpha of .88. MLQ-SF covers two facets of meaning in life: searching vs. presence [14]. As these are not identical, 5 items were used for searching for meaning in life (SMIL) and 4 items for the presence of meaning in life (PMIL) in the questionnaire. Searching for meaning gave 3 items with a Cronbach’s alpha of .775 and 3 items of presence of meaning with a Cronbach’s alpha of .796.

### Perceived stress

Five questions from A Global Measure of Perceived Stress (PSS) (Cohen et al. 1983) [15] were analyzed for internal reliability and 4 items offered a Cronbach’s alpha of .745

### Quality of work life

From the General Social Survey 2010 SECTION D Quality of Work-life Module NIOSH [16] 7 questions about quality of work life (WL) were transformed and rated in VAS format. Of these 7 items, 2 items: ‘At the place where I work, I am treated with respect’ and ‘I have often been able to control irritation in my life’ presented a Cronbach’s alpha of .764.

### Bullying

Two questions were constructed for the present purpose: Participants were asked if they were ‘bullied during training’ or had ‘seen others being bullied during their surgical training’. These 2 items gave a Cronbach’s alpha of .815.

### Statistics

The results were computed with IBM, SPSS software, version 26. One-way between-groups multivariate analysis of variance (MANOVA), as well as one-way and two-way analyses of variance between groups with Tukey’s post hoc test, were used. Scale reliability was tested for internal consistency for 6 scales used in the form of Cronbach’s alpha. Furthermore, multiple linear regression analyses, Kolmogorov-Smirnov 2-sample test (K-S), Kruskal-Wallis test (H), median test, Pearson or Spearman correlations, and independent t-tests (2-tailed) were applied when considered appropriate.

### Consent and ethical approval

Participation was voluntary and anonymous. Participants had the right to deny or disrupt their participation without consequences. By completing the questionnaire, they gave informed consent. Ethical approval was granted before the commencement of this study. The Health Research Authority and Health and Care Research Wales approved this study.

## Results

Altogether 54 surgeons completed the questionnaire; one denied, and one disrupted the participation due to work commitments. The demographics and the significant differences (ANOVA and t-tests) and correlations (Pearson’s r) between work-related issues are shown in Table 1.

**Table 1:** Participants’ demographics and results of work-related issues

Variable	Definition	N sub-group	MEAN	SD	*p*<
Demographic summary					
Age	Years	54	33.3	6.2	
Gender	Women	20			
	Men	34			
Gender and age/years	Women	17	34.4	6.7	n.s.
	Men	31	32.7	5.9	
Level of Training	JT	25	1.16	.6	
	ST	17	1.00	.0	
	CONS	12	1.75	1.4	
Work-related issues					
Quality of Work Life	JT	25	74.79	20.9	No difference between groups in the total sum of the quality of work life
	ST	17	80.24	33.5	
	CONS	12	83.50	33.7	
Bullied during training	JT	25	29.9	18.6	Consultants experienced more bullying during training 3>1, *p*= .006
	ST	17	41.4	20.2	
	CONS	12	50.3	13.0	
I do extra work besides my job (including paid or unpaid)	Yes	24			^a^Extra work correlates negatively with extra work days per month, *r*= −.389; *p=* .004
	No	29			
I work extra days per month beyond schedule ^a^	0	11			Consultants’ extra work days exceeded that of JT 3>1, *p* = .003
	1-3	22	1.92	.9	
	4-6	16	2.35	.6	
	>6	5	2.92	.9	
My usual work	Full-time	51			
	Part-time	3			
After an average work day i relax or do pleasant activities (hours)	0	4			20% relaxed 1–3 hours after work.
	1-3	44			
	>3	5			
Supervise others as a part of my job	Yes	51			
	No	3			

### Do surgeons display narcissism?

**Fig. 1:**
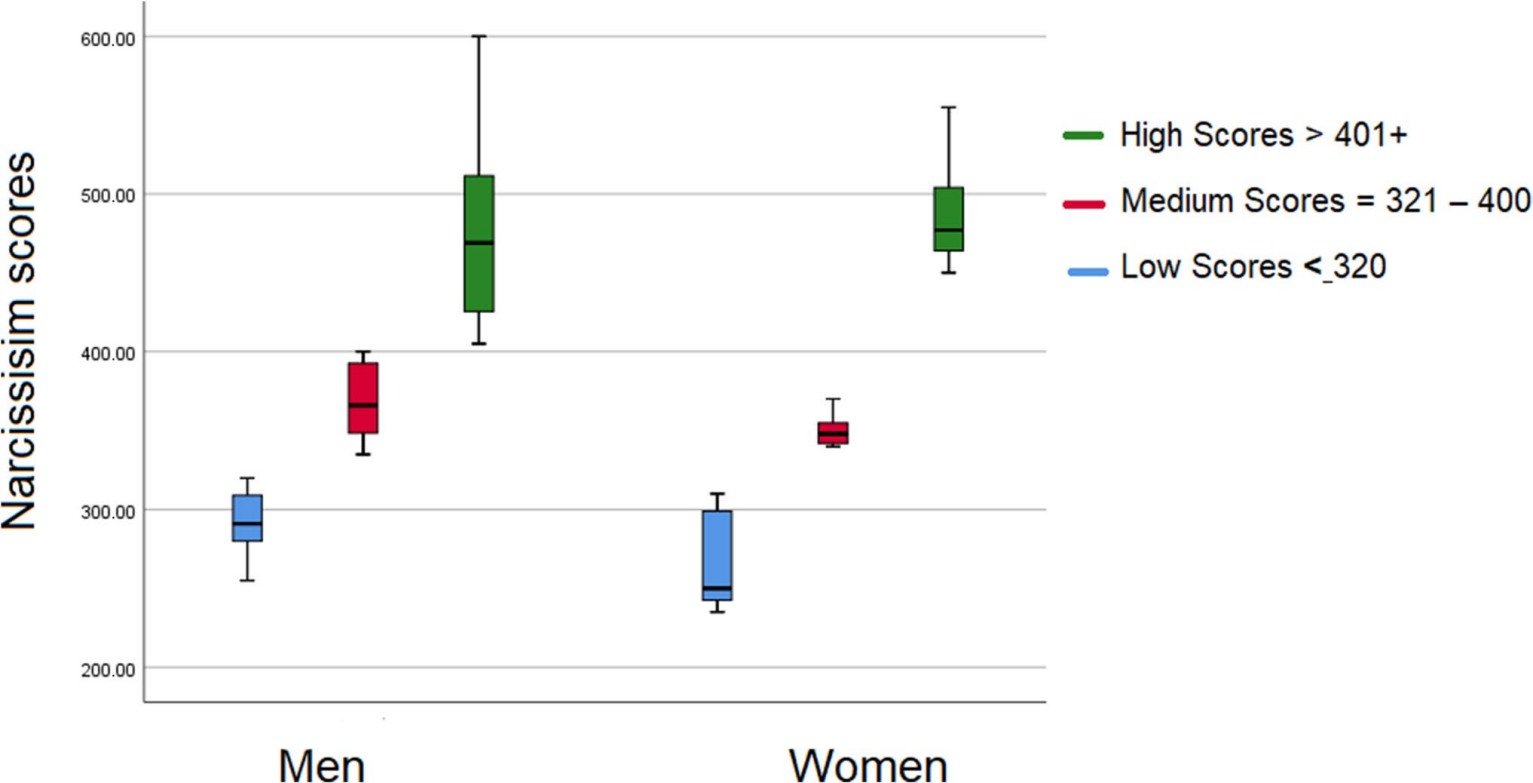
Gender differences in narcissism scores

Surgeons’ narcissism (N) was normally distributed when measured with the presently used 10-item N scale’s total sum ranging from 235 to 600, mean= 372 (SD= 89.17), and Md= 355 N-scores. K-S test for 2 samples narcissism-scores by gender was .555 (2-tailed) (Fig. 1). The N-scale was then divided into 3 groups. A total of 33% had low narcissism scores = < 320 (N-group 1); 33% presented medium narcissism scores = 321–400 (N-group 2) and 33% displayed high narcissism scores = 401–600 (N group 3). Based on this, we considered that a third of the surgeons had an inclination to display narcissism. However, out of this third, a final analysis indicated that about 22% were likely to display disruptive behaviour if challenged. No significant difference between means in men’s (M 381; SD 84) and women’s (358; SD 98) N scores was found (Fig. 1).

### Surgeons’ inclination to display narcissism between N-groups

**Fig. 2:**
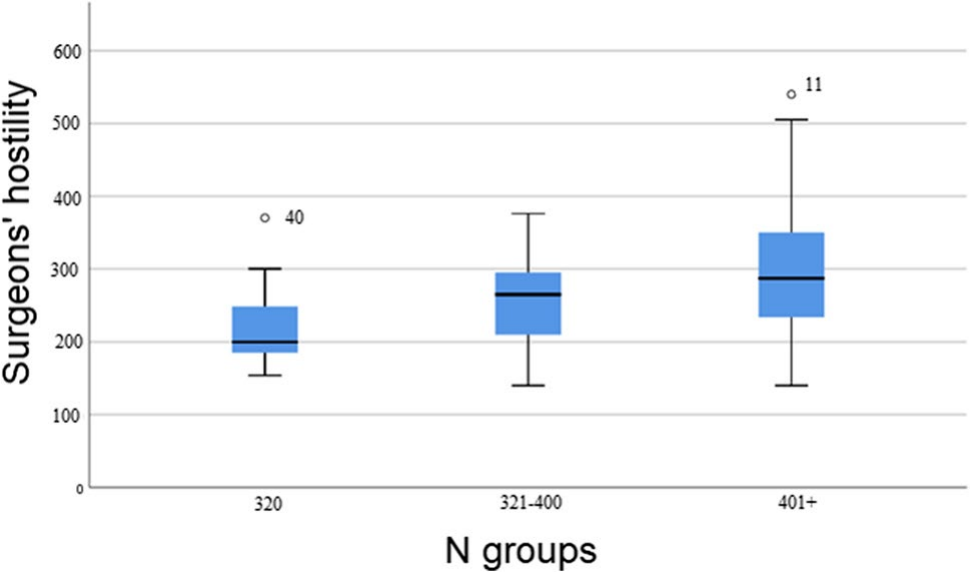
Surgeon's hostility in N groups

**Fig. 3:**
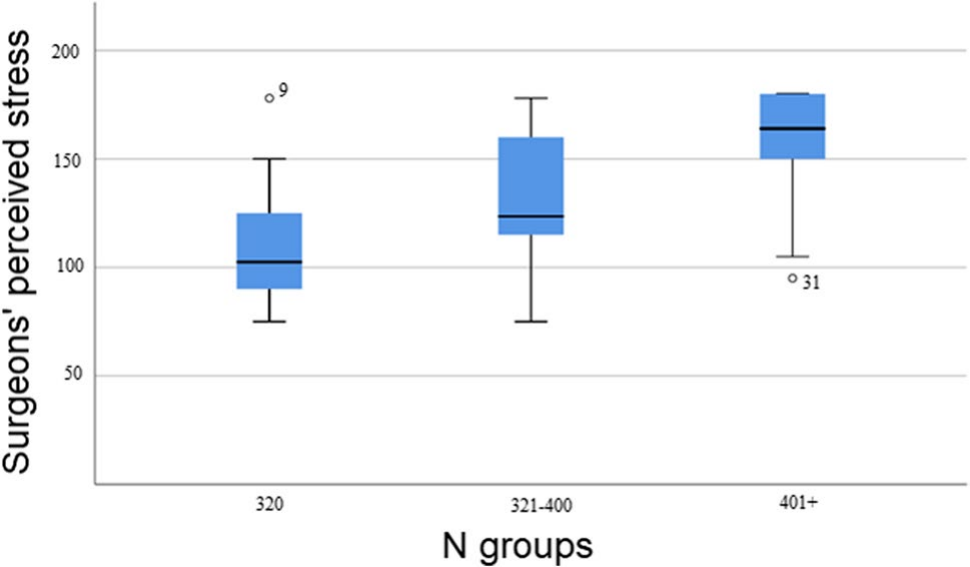
N scale’ displaying low, medium, and high narcissism scores relative to stress

A one-way between groups multivariate analysis of variance was performed to investigate differences between surgeons’ low, medium, and high inclination to display narcissism in relation to total hostility, total perceived stress, total experience of presence of meaning in life as well as of search for meaning in life. The 3 N-groups served as independent variables. Preliminary assumption testing was conducted to check for normality, linearity, univariate and multivariate outliers, homogeneity of variance-covariance matrices, and multicollinearity, with no serious violations noted. A significant difference between narcissism-groups in total hostility (*F* [2.49]=5.210; *p*<.01) was found between low and high narcissism groups (*t*[df=23.2]= 2.992; *p*<.006) (Fig. 2). The significances in total perceived stress (*F*[2.50]=13.193; *p*=.001) were revealed between low and high narcissism groups (*t*[df=32.7]=5.191; *p*=.001) as well as between low and medium narcissism-groups (*t*[df=33]=2.245; *p*=.032) (Fig. 3). The total sum of experience of *presence* of meaning in life (*F*[2.50] =3.331; *p*<.05) was found between low and high narcissism groups (*t*[df=33]= 2.665; *p*= .012). The total sum of *search *for meaning in life, did not yield any significance between groups. MANOVA’s Wilks Lambda was *p*< .001 and partial eta squared was = .78.

### Are surgeons’ N-scores reflected in hostility?

Surgeons’ hostility was scrutinized with a linear regression analysis to assess the ability of the variables ‘N scale’ (Fig. 2), ‘being bullied during training’ and ‘presence of meaning in life’ as independent variables to explain or predict the variance in the dependent variable ‘hostility’. The model was significant (*p*=.000) and its *R*^2^ explained 38.5% of the variance in total hostility. By checking each individual contributive variable’s significance, it was found that the N-scale added uniquely 23% (*p*=.000; beta= .536) followed by ‘presence of meaning in life’ with 12% (*p*=.004; beta= −.384) along with ‘being bullied during training’ with 11% (*p*=.009; beta=.325) to the explanation of the variance in hostility. In other words, N scale was the best predictor of hostility. No gender or level of training difference was found in hostility (Fig. 2).

### Are surgeons’ N-scores reflected in perceived stress?

Surgeons’ perceived stress was scrutinized with a linear regression analysis to assess the capacity of the independent variables ‘N scale’ displaying low, medium, and high narcissism scores (Fig. 3) together with ‘Seen others bullied during training’ and ’I work extra days per month beyond schedule^’^ to explain or predict the variance in the dependent variable ‘total perceived stress’. The model was significant (*p*=.000) and its *R*^2^ explained 57.1% of the variance in total perceived stress. By checking each individual contributor’s significance, it was discovered that the N-scale contributed most with 33% (beta=.570; *p*=.000) followed by ‘others were bullied during training’ with 16% (beta=.411; *p*=.000) and negatively with ‘I work extra days per month beyond schedule^’^ with 7% (beta= -.272; *p*=.007) to the explanation or prediction of the variance in surgeons’ total perceived stress. In other words, besides N-scores, observing ‘others being bullied during training’ predicts surgeons’ stress. No significant gender or level of training difference was found in surgeons’ total perceived stress. ‘I work extra days per month beyond schedule^’^ explained negatively perceived stress if the other independent variables were held constant.

### Do surgeons experience a search for meaning in life?

Surgeons’ N-groups did not differ from each other as regard ‘*search* for meaning in life’ and a linear regression analysis was done with ‘searching for meaning’ as the dependent variable to explore the power of the independent variables ‘hostility’ and ‘I do extra work besides my job’ to explain or predict this dependent variable. The model was significant (*p*=.012) and its *R*^2^ explained 16.4% of the variance in the total sum of ‘*search* for meaning in life’. Both predictors explained 8% of the variance each but ‘I do extra work besides my job’ had a higher beta value (beta .289; *p*= .032) than that of hostility (beta .278; *p*= .038) and was, therefore, a slightly better predictor of *search* for meaning in life.

### Do surgeons experience the presence of meaning in life?

**Fig. 4:**
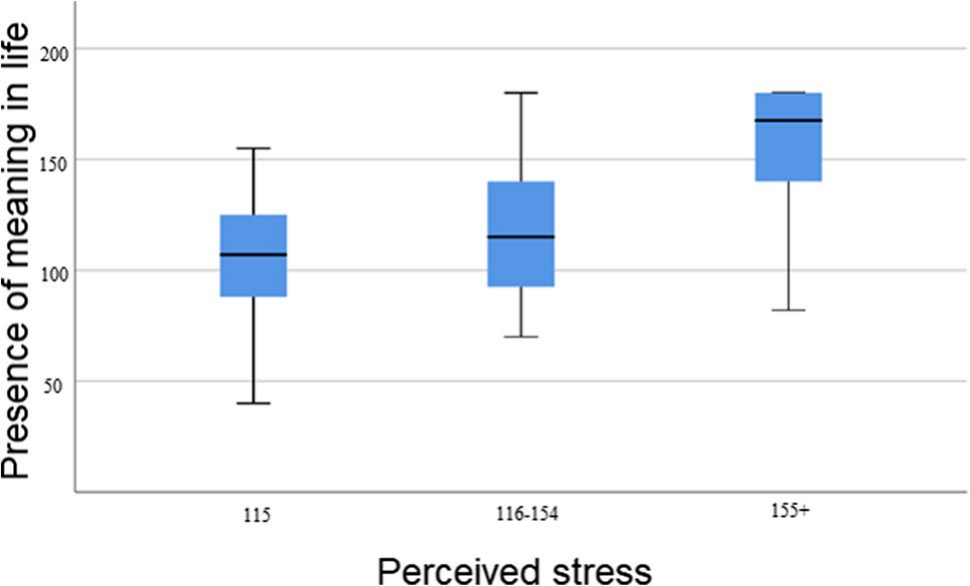
Presence of meaning in life and perceived stress

Surgeons’ N-groups did not differ from each other as regard ‘presence of meaning in life’; a linear regression analysis was done with ‘presence of meaning in life’ as a dependent variable to explore the capacity of the independent variables ‘age’, ‘hostility’, ‘I do extra work besides my job’ and ‘perceived stress’ to explain this dependent variable. The model was significant (*p*=.000) and its *R*^2^ explained 62 % of the variance in total ‘presence of meaning in life’. The individual significant predictors ‘perceived stress’ explained at 48% (beta .751; *p*= .000) and ‘age’ at 5% (beta .231; *p*= .022) and negatively ‘I do extra work besides my job’ at 6% (beta −.246; *p*= .016) and hostility 10% (beta −.315; *p*= .003). When the beta value was negative, there was negative correlation between the dependent variable and the corresponding independent variable if the other independent variables were held constant. Furthermore, between surgeons’ presence of meaning in life by low, medium and high perceived stress- groups two significant differences were found: between low and high [*p* = .000] and medium and high [*p *= .002]) stress groups. Surgeons with high level of stress experienced most presence of meaning in life. (Fig. 4).

### Does surgeons’ level of training reflect in the experience of the presence of meaning in life?

**Fig. 5:**
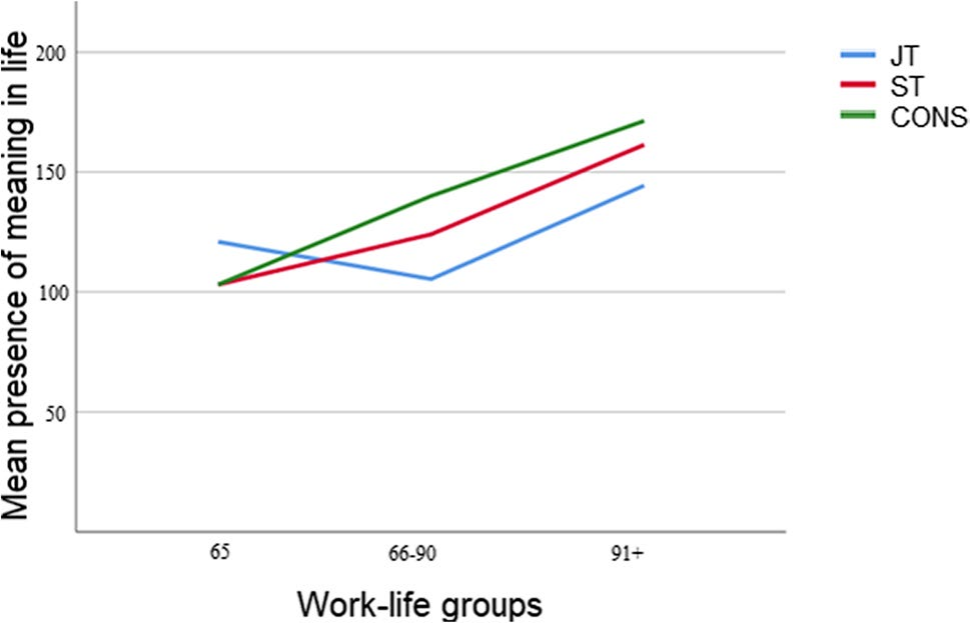
Mean presence of meaning in life in work-life groups

**Fig. 6:**
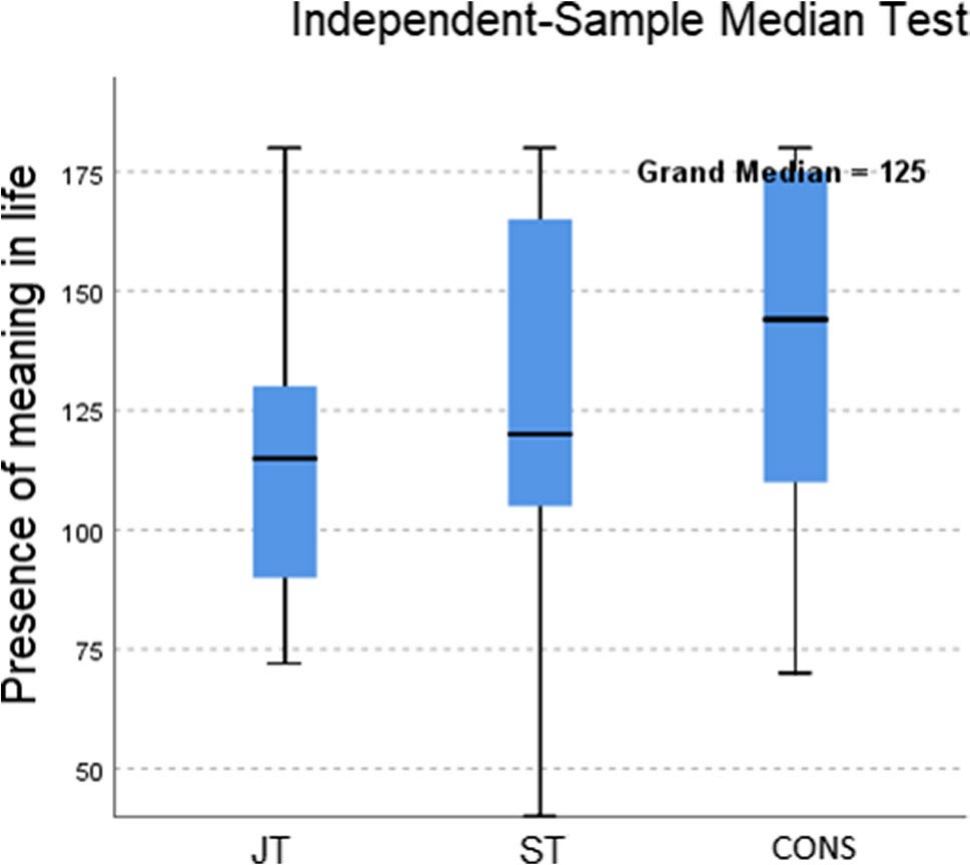
Presence of meaning in life in JT, ST, and CONS

To take a closer look at surgeons’ level of training and its relation to ‘presence of meaning in life’, a nonparametric Median test was performed. Between JT and CONS medians in ‘presence of meaning in life’, a significance was found (*p*= .045) In other words, As regards work-life groups a 2-way ANOVA indicated that between work-life groups and ‘presence of meaning in life’ a significance (F[2.52]=11.39; p= .001) between low < 65 and high 91+ was at hand. Training was n.s. betweenwork-life groups. Surgeons’ presence of meaning in life was analyzed by Median test for difference between levels of training. JT vs CONS (= 6.036; Bonferroni adjusted: p= .045). CONS experienced more presence of meaning in life than JT (Figs. 5 and 6).

### Bullying during surgical training

Surgeons’ total bullying by training was significant (H [2] =10.67 = *p* =.005). A closer look displayed that the “experience of being bullied during training” by training was significant (H [2]= 8.50 = *p* =.014). High N-group members experienced being bullying during training (M [13]= 106.7; SD 17.57) more so than members of low N-group (M[26]=72.4; SD 31.74). In other words, JT experienced less bullying than CONS. When looking at those who had seen others being bullied during surgical training, it was found that between JT and CONS, the latter had seen more ‘others being bullied during training’ (M [13]= 53.4; SD 9.1) than JT (M[26]=41.5; SD 16.4; *p*=.001).

### Summary of results

Based on surgeons’ displayed N-scores, a third of them exhibited an inclination to demonstrate narcissistic behaviour. This third decreased to 22% of the surgeons in a final analysis and these surgeons were thought to display disruptive behaviour if challenged. Significant differences between surgeons with low, medium, and high N-scores were found in hostility and perceived stress. The N-scale was the best predictor of hostility; also ‘being bullied during training’ contributed significantly to hostility. Likewise, surgeons’ perceived stress was predicted most by N-scale followed by observing that ‘others were bullied during training’ but was explained negatively by the variable ‘I work extra days per month beyond schedule’ in the prediction of the variance in surgeons’ total perceived stress. Nevertheless, ‘stress’ was the best predictor of the experience of the *presence* of meaning in life. Yet, ‘I do extra work besides my job’ predicted ‘*search* for meaning in life’ but explained negatively ‘*presence* of meaning in life’. Compatibly, ‘hostility’ predicted ‘*search* for meaning in life’ but explained negatively ‘*presence* of meaning in life’. In other words, surgeons can experience both forms of meaning in life but not simultaneously.

## Discussion

### Hostile work environment

The frequency of hostile work environments in surgery has not been well-studied or reported; therefore, it is problematic to identify and address mistreatment and hostility in the workplace within the commonly established surgical culture. Efforts to identify and address workplace hostility are fundamental to enhance the accomplishment of the academic surgical environment [17]. We found that 33 % of the surgeons displayed narcissistic tendencies in the forms of scoring higher on the presently used N-scale but 22% were at risk for displaying disruptive behaviour. Our results agreed with those revealed by previous researchers [18] who discovered that although both narcissism and hostility mirrored an unkind, unfriendly interpersonal style, they were undeniably different personality traits. Our results from MANOVA indicated that there was a significant difference between low and high-scoring N-groups as regards total hostility. Furthermore, our regression analysis of hostility indicated that the N-scale was its best predictor.

### Narcissism score and hostility

For that reason, N-scores are reflected in hostility. Yet, also ‘being bullied during surgical training’ was a significant predictor of hostility. A previous study [19] analyzed the relation between narcissism and hostility which ‘was significant for males and females, for people of all ages, for students and nonstudents, and for people from individualistic and collectivistic countries’. Reaction to provocation was the reason for hostility. In agreement, our surgeons scoring high in N-groups were people disposed to hostility.

### Perceived stress and work environment

We found that N-scores are reflected in surgeons’ perceptions of stress. Many surgeons believe that they are more stress resilient than their colleagues in other specialties. The traits that used to define them in the forms of commitment, self-sacrifice, and singularity of focus, may today put surgeons at risk for a too big workload and for an imbalance between personal and professional life [20]. Namely, a national survey of 1000 participants showed that surgeons’ mean scores on free-floating and hysterical-anxiety subscales of the mental health index were significantly higher than those of the general population in the UK [21]. Consequently, we performed a regression analysis predicting surgeons’ total perceived stress. We found that the N-scale contributed most to the stress followed by ‘seeing others bullied during training’. In contrast, ‘I work extra days per month beyond schedule^’^ was a negative predictor of the variance in surgeons’ total perceived stress. It seemed like surgeons were satisfied with working extra and not consciously aware of the risks of doing too much work. Yet, professional responsibilities make maintaining a work-life balance difficult and could impair physical and mental-health aftermath due to being exclusively career oriented [22]. Currently, surgeons’ extra work was not experienced in a negative way in their perceived stress. Working extra is rewarded with money and therefore experienced as beneficial stress.

### Experience bullying

High N-group members experienced bullying during training more than low N-group. In other words, JTs experienced less bullying than consultants. When looking at those who had seen others being bullied during surgical training, it was found that CONS had seen more ‘others being bullied during training’ than JT. Surgeons are subject to bullying during surgical training; yet, previous research [23] studied who becomes a bullying perpetrator after the experience of being a bullied victim and referred to the threatened egotism theory [24]. This means that one’s highly favorable view of self is challenged by some person or circumstance. Exaggerated, unstable, or insecure beliefs in the self’s superiority meets threats and is causing violence [23]. It has been exposed that the relationship between prior bullying victimization and subsequent bullying perpetration was moderated by self-esteem [23]. Higher self-esteem persons were the most likely to engage in future bullying perpetration in response to bullying victimization, while those with lower self-esteem were the least likely to engage in future bullying perpetration. According to Choi and Park’s theory, 22% of our surgeons could exhibit disruptive behavior if challenged [23]. Yet**, **it is understandable that the JT group experiences more insecurity and lower self-esteem compared to the more experienced surgeons in the CONS group. Theoretically, the JT group would be less prone to engage in bullying perpetration later on. This insight could also initiate an improvement in the current surgical environment compared to the old generation of surgeons.

### Search for meaning in life

It was previously claimed that a person’s “search for meaning is the primary motivation in his life and not a ‘secondary rationalization’ of instinctual drives [25]. This meaning is unique and specific in that it must and can be fulfilled by them alone; only then does it fulfill a significance that will satisfy their own will to the meaning. Surgeons are now and again faced with the unsuccessful efforts. As they cannot talk safely about the emotional impact of their work, they may harbor the pressure of their work beyond the operation theatre, placing them at risk of depression, substance misuse, and even suicide [26]. Searching or pursuing meaning in life does not mean that a presence of meaning is found; namely, the former relates to personality traits in a more negative way [14] in agreement with our results where hostility explained *search for *meaning but predicted negatively *presence* of meaning. In contrast, a hyper-intention for *searching* hampers the experience of the *presence* of meaning in life; the latter is a byproduct when focusing on one’s unique gifts and presently quality of work life [25].

### Presence of meaning in life

Surgeons’ N-groups did not differ from each other as regards the ‘presence of meaning in life’; our regression analysis indicated that ‘perceived stress’ was the best predictor of experiencing the presence of meaning in life. We interpreted that the stressed surgeons were mostly subject to beneficial or rewarded stress which can be beneficial and is actually necessary for overall well-being. This type of "positive" stress contributes to feelings of optimism and excitement about life. It is *de facto* contributing to a healthy life. Medical challenges can be stimulating and even pleasurable to conquer. However, ‘I do extra work besides my job’ as well as ‘hostility’ predicted negatively surgeons’ ‘presence of meaning in life’ and then their stress was likely to turn into distress [27]. By taking a closer look at surgeons’ ‘presence of meaning in life’ by the level of training it was revealed that CONS experienced more presence of meaning in life than JT group. Yet, ‘we ourselves may have a different life purpose at each stage of life. The important thing is for each goal to give us satisfaction and encouragement to get up in the morning and fight for what we want’ [25].

### Limitations

There was a challenge to collect the anonymous data and recruit the participants during their busy schedule as surgeons and for them to complete the questions for demographic data plus 52 questionnaires from 2 different busy UK hospitals. The use of our short version comprising 10 items from NPI as a measure of narcissism, in a sample consisting only of surgeons, made us abstain from external generalizing beyond normally distributed character variations to any conclusions about diagnosable NPD [28]. The use of scales requiring self-reports means that people are often biased by their own experiences or by illusions of others’ expectations and it is sometimes difficult to control any exaggerations.

### Implications

Disruptive behaviour exist among surgeons; trainees might fear the consequences of reporting it, therefore, encouragement and support must be available [29]. Education in the surgical environment is fundamental for improving the surgical atmosphere. Therefore, surgical associations are challenged to work on improving awareness and provide mental health support to promote excellence in surgical training. Such an improvement benefits both patients and trainees. In addition, National Health System hospitals in the UK have been encouraged to appoint Freedom to Speak Up Guardians to ensure a healthy speaking-up culture. The data can be replicated in the future in multicentered international settings to study surgeons’ personality traits in relation to the work environment.

## Conclusion

Altogether 22% of the surgeons were likely to exhibit disruptive behaviour in a challenging situation. The N-scale was the best predictor of hostility and perceived stress. Being bullied during surgical training predicted hostility. Seeing others being bullied during surgical training predicted stress as opposed to working *extra days per month* beyond the schedule. The latter seemed to be beneficial stress while rewarded with extra money. Namely, stress was the best predictor of the experience of *presence* of meaning in life. Hostility’ contributed to ‘*search* for meaning in life’ but explained negatively ‘*presence* of meaning in life’. In other words, search and presence of meaning in life are two unique concepts; ‘search’ does not automatically lead to ‘presence’ of meaning. Consequently, surgeons can implicitly experience both forms of meaning in life but explicitly only one form at a time.

## Declarations

### Dedication

We would like to dedicate this paper to the soul of the dear friend and colleague, Mr Chekwas Obasi. May your soul rest in peace and power.
